# Roux‐en‐Y procedure to reconstruct the upper gastrointestinal tract in six dogs and five cats: A descriptive case series

**DOI:** 10.1111/vsu.14259

**Published:** 2025-04-01

**Authors:** Brent Fink, Sarah Marvel, Eric Monnet

**Affiliations:** ^1^ Department of Clinical Sciences, College of Veterinary Medicine and Biomedical Science Colorado State University Fort Collins Colorado USA

## Abstract

**Objective:**

To describe the Roux‐en‐Y principle for upper gastrointestinal reconstruction and to report its outcome in dogs and cats.

**Study design:**

Retrospective study.

**Animals:**

A total of 11 client‐owned pets (six dogs and five cats).

**Methods:**

Medical records of dogs and cats treated with Roux‐en‐Y principles were reviewed.

**Results:**

Biliary obstruction was diagnosed in six cases, and upper gastrointestinal obstruction in five cases. Biliary carcinoma was the most common histological diagnosis. All the procedures were successfully completed. One dog and one cat died of cardiac arrest one and 5 days after surgery, respectively. Another cat was euthanized because of septic peritonitis 6 days after surgery. One dog died 11 days after surgery because of portal vein thrombosis and septic peritonitis. Another dog was euthanized because of septic peritonitis 6 days after surgery. Gastrostomy tubes were used in six animals. Gastric dilatation, ileus, and vomiting required medical attention after surgery. The median survival time was 82 days (range: 60–196 days) for the four dogs and 365 days (range: 84–410 days) for the three cats discharged from the hospital. The median survival time of animals diagnosed with neoplastic and non‐neoplastic disease was 5 days (range: 1–196 days) and 192 days (range: 6–410 days), respectively.

**Conclusion:**

The Roux‐en‐Y principle could be considered to treat biliary and upper gastrointestinal obstructions in dogs and cats. The underlying disease greatly influenced the outcome.

**Clinical significance:**

The Roux‐en‐Y principle could be used as an alternative to cholecystoduodenostomy or Billroth II.

## 1 INTRODUCTION

Indications for upper gastrointestinal reconstruction include gastric neoplasia, pyloric outflow obstructions, pyloric perforations, proximal duodenal disease, and biliary disease. Pyloroplasty, Billroth I, Billroth II, and cholecystoduodenostomy with or without a Billroth II procedure are traditional reconstruction techniques used in dogs and cats.^1^, ^2^, ^3^ Billroth II is associated with the potential for biliary reflux into the stomach, afferent loop syndrome, and dumping syndrome in human patients.^4^ In upper gastrointestinal reconstruction procedures, the afferent loop refers to the segment of the duodenum and proximal jejunum that lies upstream from a gastrojejunostomy. This afferent loop transports pancreatic fluid and bile toward the gastrojejunostomy. Afferent loop syndrome occurs due to an obstruction of flow in this segment leading to an accumulation of pancreatic fluid and bile.^4^ This obstruction of flow can result from decreased motility, kinking of the segment, or stenosis at the anastomosis of the afferent loop to the stomach which may predispose this segment to bacterial overgrowth.^4^ The dumping syndrome results from the fast emptying of the stomach content directly into the jejunum.^4^ Both afferent loop syndrome and dumping syndrome can result in nausea and discomfort, leading to vomiting and anorexia.^4^ Cholecystoduodenostomy, which is often performed concurrently with the Billroth II procedure, can be associated with reflux of duodenal content into the gall bladder, resulting in chronic cholangiohepatitis.^5^, ^6^, ^7^ Together, these complications can lead to weight loss, poor recovery, and a worse long‐term prognosis.

The Roux‐en‐Y principle or technique interposes a loop of jejunum between the stomach and the proximal jejunum or between the gallbladder and another segment of the small intestine (duodenum or jejunum). This allows digestive secretions from the gallbladder and pancreas to mix with digesta aborad to the stomach which is meant to prevent bile reflux into the stomach or reflux of gastrointestinal contents into the gallbladder.^4^ In humans, the Roux‐en‐Y procedure has been shown to provide an advantage over Billroth II reconstruction techniques in that it reduces the incidence of post‐gastrectomy syndromes, including afferent loop syndromes, dumping syndromes, and stoma ulceration.^4^, ^8^ Dogs have been used in different experiments to evaluate the effect of the Roux‐en‐Y on gastrointestinal function^9^, ^10^, ^11^; however, after conducting a literature search on PubMed in 2024, there is no report in the literature about the Roux‐en‐Y used to treat client‐owned dogs. The decision to use the Roux‐en‐Y principle in this case series was based on negative experiences with the Billroth II procedure.

The objectives of this case series were to describe the Roux‐en‐Y principle for upper gastrointestinal reconstruction and to report its outcome in dogs and cats.

## 2 MATERIALS AND METHODS

Digital medical records from the Colorado State University Veterinary Teaching Hospital were reviewed to identify dogs and cats treated with a Roux‐en‐Y procedure since the first instance of the surgery in 2016.

Data collected from the medical record included signalment, clinical signs at the time of presentation, duration of clinical signs before surgery, medical treatment, and blood work performed before and after surgery. Radiographs and ultrasound images performed before surgery were reviewed. Information on the procedures performed on each patient was collected from surgical reports. Postoperative treatment was reviewed from the critical care medical record.

Long‐term follow‐up information was collected from either the medical records at our institution, phone calls with the owners, or the referring veterinarians. Cause of death was recorded as related to the surgical procedure (development of septic peritonitis, biliary obstruction, development of gastrointestinal ulceration), or unrelated to the surgical procedure if it was due to the underlying disease or other disease.

Descriptive statistics were used to describe the study population. Continuous data are reported as median and range. JMP (JMP 17, SAS, Cary, North Carolina) was used for the statistical analysis.

## 3 RESULTS

### 3.1 Description of the population

Six spayed female dogs, three spayed‐female cats and two male castrated cats were included in the study. The median age was 11 years (range: 2–16.1 years) for the dogs and 10 years (range: 7–12 years) for the cats. Dog breeds included a Doberman, Golden Retriever, Vizsla, Cocker Spaniel, Bull Mastiff, and an Australian Kelpi mix. The feline population included three domestic short hairs, one domestic medium hair, and one domestic long hair.

### 3.2 Clinical presentation and diagnosis

Anorexia was the most common complaint at the time of presentation, along with vomiting in six dogs and four cats. Icterus was documented in three cats and two dogs at the time of presentation time (Table 1).

**TABLE 1:** Results of complete blood counts and biochemistry for the 11 cases treated with a Roux‐en‐Y procedure.

Parameter	Dogs		Cats	
	Before surgery	After surgery	Before surgery	After surgery
BUN (mg/dL)	11 (9–26)	11 (6–36)	23 (14–27)	26 (12–29)
Creatinine (mg/dL)	1 (0.3–1.2)	0.53 (0.40–2.50)	1.2 (1–1.5)	0.90 (0.70–1.30)
ALP (IU/L)	798 (25–3349)	385 (59–2418)	119 (33–938)	45 (33–457)
ALT (IU/L)	233 (312640)	361 (32–592)	832 (85–1000)	212 (59–433)
Bilirubin (mg/dL)	0.8 (0.1–11.1)	0.70 (0.10–6.10)	3.7 (0.10–10.6)	0.70 (0.10–1.20)
Protein (g/dL)	5.3 (3.1–6.4)	4.55 (3.50–4.90)	7.2 (4.3–9.0)	5.20 (4.30–6.0)
Albumin (g/dL)	2.7 (1.6–3.2)	2.20 (1.90–2.60)	2.6 (2.1–3.5)	2.50 (2.0–3.30)
Neutrophils (10^3^ × μL)	12.9 (6.5 22.9)	18.3 (8.10–38.4)	4.1 (3.45–10.7)	7.7 (5.40–12.0)
Lymphocytes (10^3^ × μL)	1.4 (0.2 4.3)	1.15 (0.40–3.80)	0.8 (0.4–3.4)	1.20 (0.60–1.50)
Platelets (10^3^ × μL)	222 (32–801)	164 (29–860)	411 (177–516)	164 (29–860)

*Note*: Blood work was performed while dogs and cats were still in the hospital.

Abbreviations: ALP, alkaline phosphatase; ALT, alanine transaminase; BUN, blood urea nitrogen; IU, international units (median and range).

The median length of medical management before surgery was 304 days (range: 1 day–4 years), with six animals failing medical management within 14 days. Six cases were treated for biliary disease consisting of obstruction of the common bile duct, and five cases were treated for gastrointestinal disease. Dog #6 had a history of intrahepatic shunt banded with thin film. This dog later developed a proximal duodenal perforation that was primarily repaired 1 year after shunt attenuation. One year later, the dog experienced another gastrointestinal obstruction due to adhesions and subsequent stricture at the previous surgical site. A Roux‐en‐Y procedure was utilized to bypass the obstruction in this case because of the decreased access and mobility to the pylorus and proximal duodenum secondary to the severe adhesions.

Biopsy of the liver and pancreas were collected as needed. Carcinoma including neuroendocrine carcinoma of the biliary tract was the most common neoplasia diagnosed (Table 2). Complete resection was not achieved in any case because of the diffuse nature of the disease.

**TABLE 2:** Surgical findings, histopathology, surgical techniques performed and outcome of six dogs and five cats treated with a Roux‐en‐Y procedure.

Case	Surgical findings	Histology	Surgical procedure	Surgical procedure for reconstruction	Status	Cause of death	Follow up time
Cat #1	Biliary obstruction Mass	Cholangiohepatitis	Resection distal CBD with mass, gastrostomy tube	Cholecystojejunostomy and jejunoduodenostomy. Figure 2	Dead	Septic bile peritonitis	410 days
Cat #2	Biliary obstruction	Pancreatitis and cholangiohepatitis	Choledochotomy	Choledochojejunostomy and jejunojejunostomy. Figure 4	Alive	NA	155 days
Cat #3	Diffuse duodenal thickening	Gastrointestinal eosinophilic sclerosis fibroplasia	Billroth 1, CBD ligated, gastrostomy tube, jejunostomy tube	First surgery: Cholecystojejunostomy and jejunojejunostomy. Figure 2	Dead	Septic peritonitis	6 days
				Second surgery: Abdominal exploration. No dehiscence. Euthanasia at owner request			
Cat #4	Mass in common bile duct with diffuse increased thickness of the CBD	CBD carcinoma and cholangiofibrosis	Resection distal CBD	Choledochojejunostomy and jejunojejunostomy. Figure 4	Dead	Cardiac arrest	5 days
Cat #5	Severe dilation of biliary system	Congenital ductal plate malformation	Drainage of biliary system	Choledochojejunostomy and jejunojejunostomy. Figure 4	Alive	NA	84 days
Dog #1	Proximal duodenal mass	Carcinoma	Gastroduodenal resection, CBD intact, J through G tube	Gastrojejunostomy and jejunojejunostomy. Figure 1	Dead	Cardiac arrest	1 day
Dog #2	Mass at duodenal papilla	Neuroendocrine carcinoma	Resection distal CBD, J through G tube	Choledochojejunostomy and jejunojejunostomy. Figure 4	Dead	Necrotizing cholecystitis	196 days
Dog #3	Pyloric hypertrophy	NA	Gastroduodenal resection, CBD intact, J through G tube	Gastrojejunostomy and jejunojejunostomy. Figure 3	Dead	Biliary obstruction Hepatitis	82 days
Dog #4	Liver mass with obstruction of CBD	Neuroendocrine carcinoma	Biopsy of liver	First surgery: Choledochojejunostomy and jejunojejunostomy. Figure 4	Dead	Dehiscence	11 days
						Septic peritonitis	
				Second surgery: Revision of distal anastomosis because of dehiscence			
Dog #5	Mass proximal duodenum obstructing duodenal papilla	Large cell lymphoma	Billroth 1, CBD ligated, Gastrostomy tube, jejunostomy tube	Cholecystojejunostomy and jejunojejunostomy. Figure 4	Lost follow up	NA	4 days
Dog #6	Stenosis and plication of the proximal duodenum	NA	Abdominal exploration	First surgery: Gastrojejunostomy and jejunojejunostomy. Figure 3	Dead	Perforating ulcer	60 days
						Septic peritonitis	
				Second surgery: Gastrojejunostomy and jejunojejunostomy because of dehiscence of distal jejunojejunostomy			

*Note*: If a case had two surgeries they are listed as first surgery and second surgery with the surgical finding at the second surgery. Cases are listed in chronological order for cats and dogs.

Abbreviations: CBD, common bile duct; G, gastrostomy; J, jejunostomy; NA, not available.

### 3.3 Surgical procedures

**FIGURE 1:**
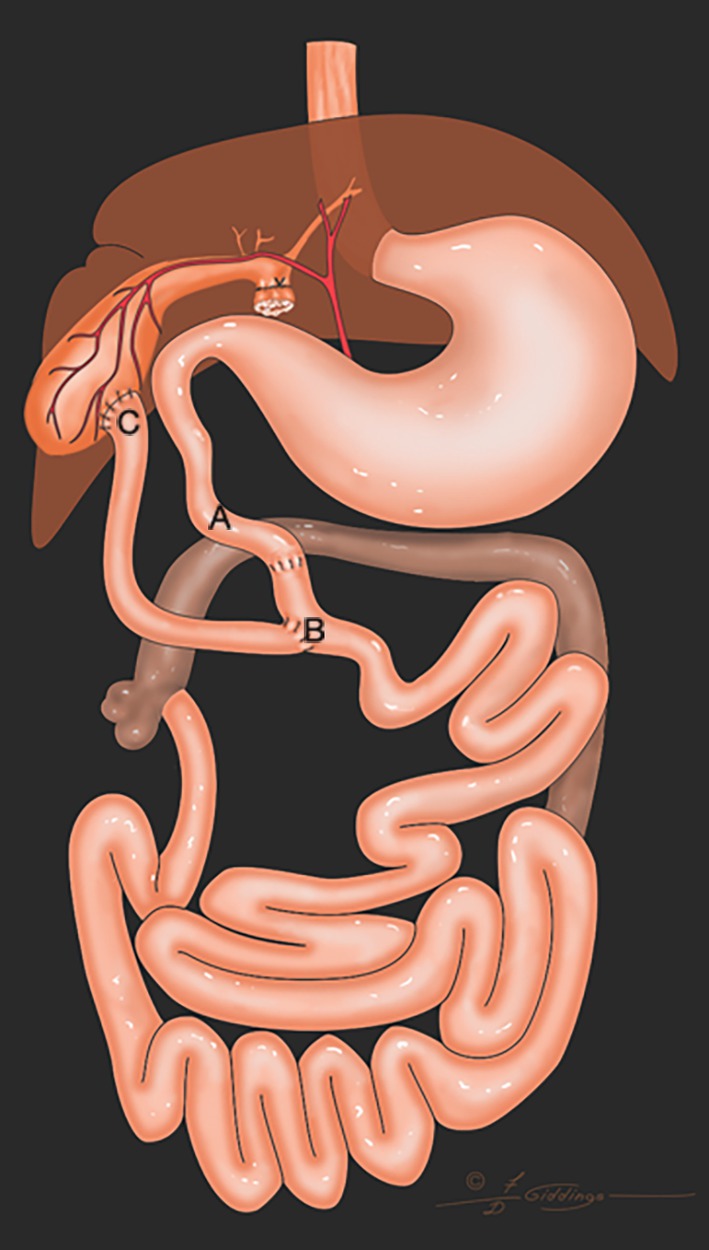
The distal segment of the common bile duct was resected. A loop of jejunum was isolated on its pedicle from the proximal jejunum, and the proximal end of the segment was brought to the gallbladder to complete a cholecystojejunostomy. An end‐to‐end anastomosis was completed, where the isolated loop of jejunum was harvested. An end‐to‐side anastomosis of the proximal jejunum was completed to re‐establish the continuity of the jejunum. A, duodenocolic curvature; B, end to side jejunojejunostomy; C, cholecystojejunostomy. Distance AB <5 cm; Distance CB: 15–20 cm.

**FIGURE 2:**
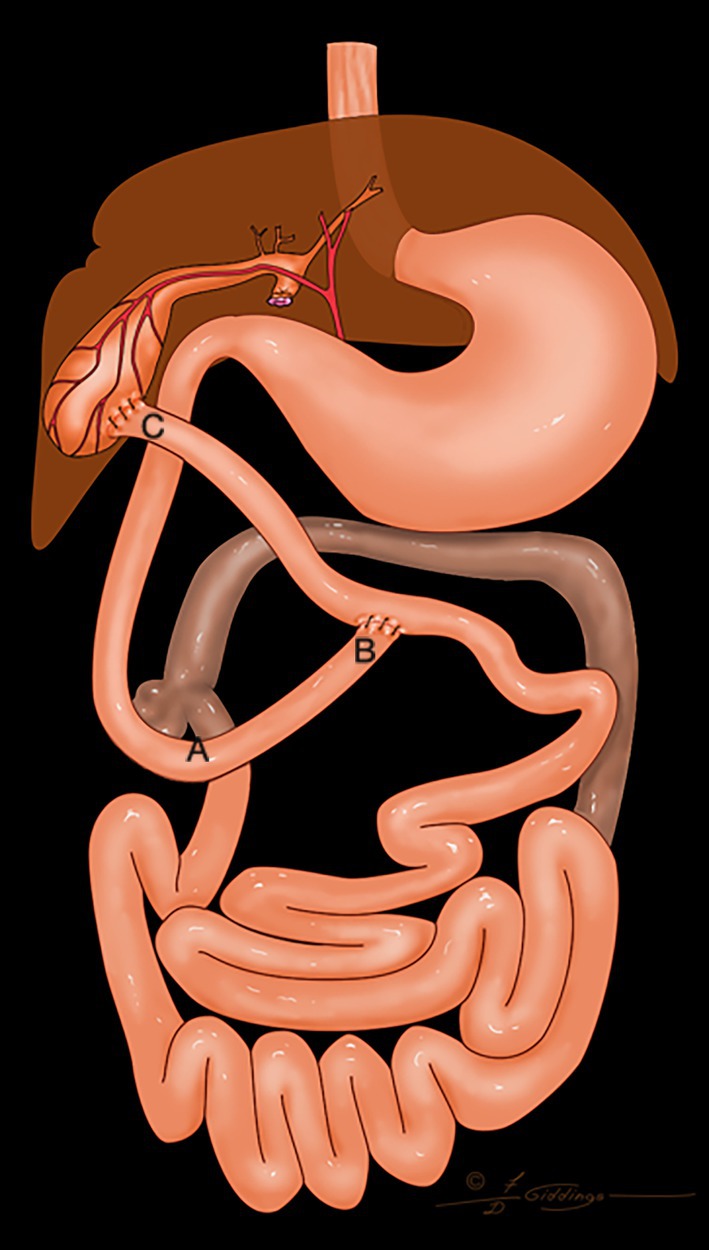
The distal segment of the common bile duct was resected. The proximal jejunum was transected, and the distal segment of the jejunum was brought to the gallbladder to complete a cholecystojejunostomy. An end‐to‐side anastomosis of the proximal jejunum was completed to re‐establish the continuity of the jejunum. A, duodenocolic curvature; B, end to side jejunojejunostomy; C, cholecystojejunostomy. Distance AB <5 cm; Distance CB: 15–20 cm.

**FIGURE 3:**
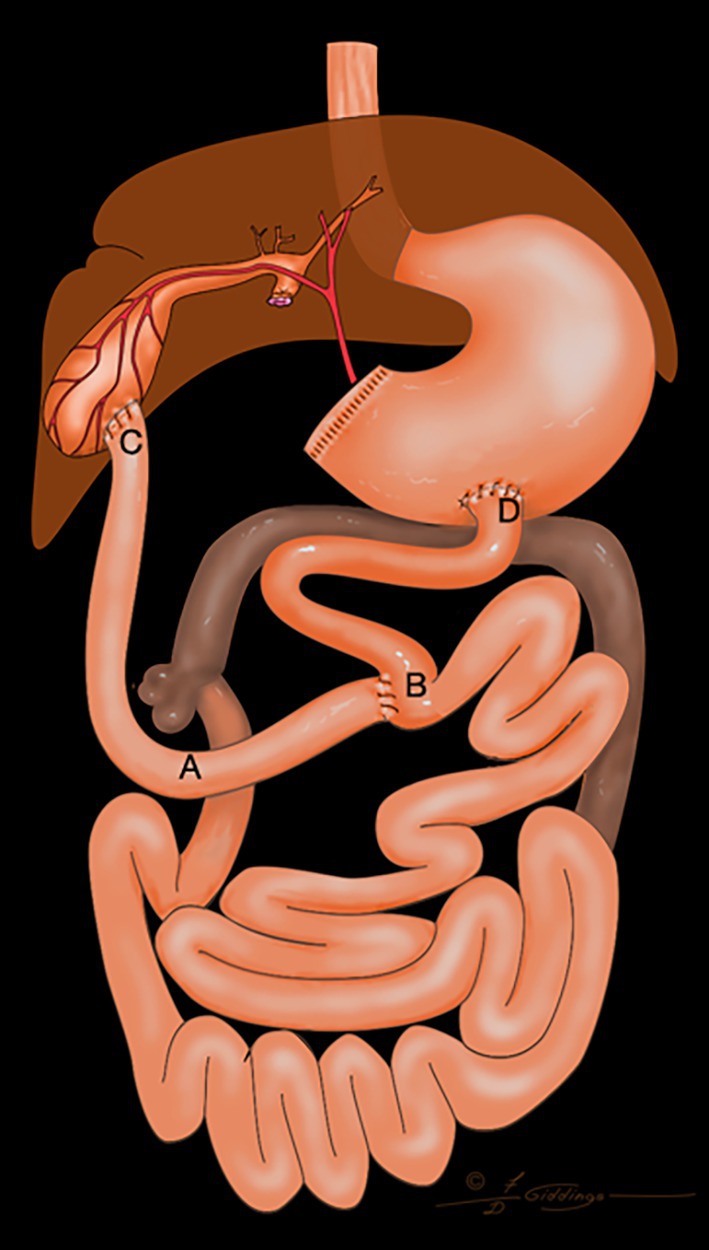
The pyloric antrum and proximal duodenum were resected, involving the duodenal papilla. A cholecystoduodenostomy was completed. The proximal jejunum was transected, and the distal segment was anastomosed to the stomach. An end‐to‐side anastomosis of the proximal jejunum was completed to re‐establish continuity of the jejunum. A, duodenocolic curvature; B, end to side jejunojejunostomy; C, cholecystoduodenostomy; D, gastrojejunosotmy Distance AB <5 cm; Distance CD: 15–20 cm.

**FIGURE 4:**
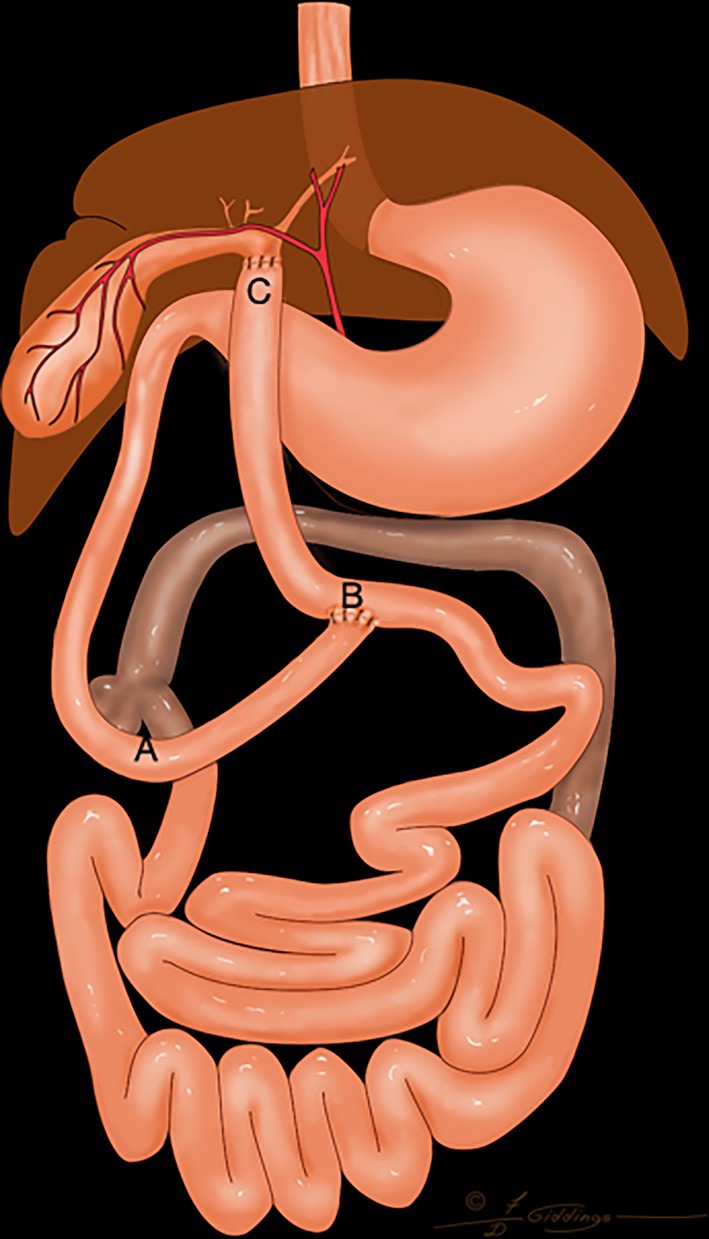
The distal segment of the common bile duct was resected. The proximal jejunum was transected, and the distal segment of the jejunum was brought to the dilated common bile duct to complete a choledochojejunostomy. An end to side anastomosis of the proximal jejunum was completed to reestablish continuity of the jejunum. A, duodenocolic curvature; B, end to side jejunojejunostomy; C, choledochojejunostomy. Distance AB <5 cm; Distance CB: 15–20 cm.

**FIGURE 5:**
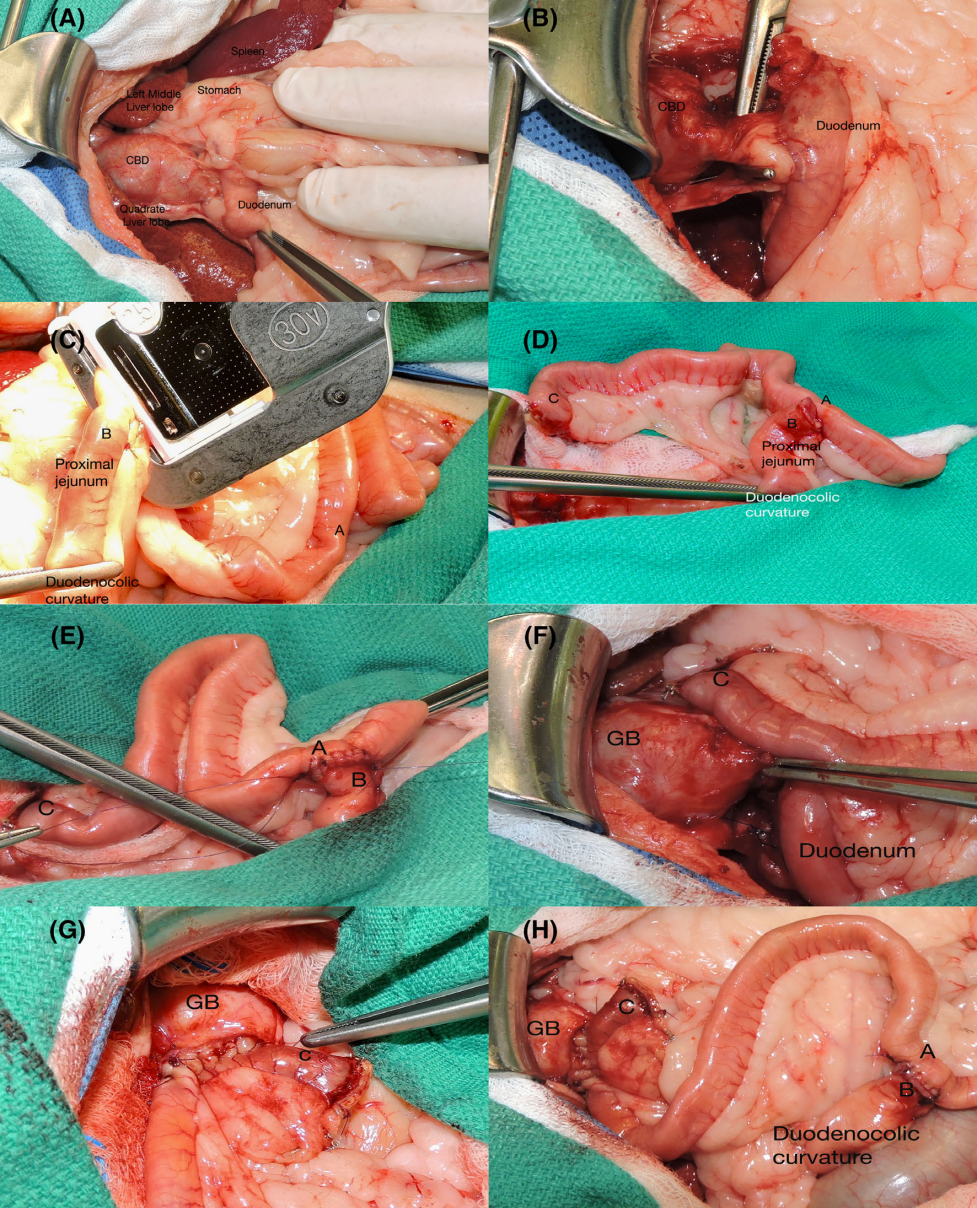
This case illustrates the technique reported in Figure 2. (A) The common bile duct is invaded by a neuroendocrine carcinoma. (B) A subjectively normal‐looking segment of the common bile duct was identified close to the duodenum. It was ligated with a 4–0 monofilament nonabsorbable suture end divided. The common bile duct was resected close to the cystic duct. (C) The limb of the jejunum is isolated for the Roux‐en‐Y. A thoracoabdominal stapler is used to close the distal part of the jejunum that will be moved to the gallbladder for a cholecystoduodenostomy. A Doyen clamp was placed on the proximal jejunum to minimize contamination. (D) The limb of the jejunum is isolated, and one jejunal artery has been ligated to improve mobilization. (E) The jejuno‐jejunostomy has been completed as an end‐to‐side anastomosis with two simple continuous suture patterns with 4–0 glycomer 631 (Biosyn, Medtronic). (F) The proximal end of the limb of the jejunum for the Roux‐en‐Y has been moved close to the distended gallbladder. (G) The cholecystojejunostomy has been completed with two simple continuous suture patterns with 4–0 glycomer 631. (H) The Roux‐en‐Y is completed. CBD, common bile duct; GB, gallbladder; A, site of the future end to side jejunojejunostomy; B, proximal jejunum less than 5 cm from the duodenocolic curvature; C, proximal end of the limb of jejunum that will be used to perform the cholecystojejunostomy. Distance from A to C between 15 and 20 cm.

A total of 10 surgeries were performed by the same board‐certified surgeon assisted by a surgery resident. One surgery was performed by another board‐certified surgeon familiar with the procedure. The Roux‐en‐Y principle was applied in four different types of procedures to achieve decompression of the biliary tract or palliate upper gastrointestinal obstruction (Table 2 and Figures 1, 2, 3, 4). When the bile duct was diseased it was ligated and transected through subjectively normal‐looking tissue close to the duodenum (Figure 5A,B). When the proximal duodenum had to be resected, a pancreatectomy was not required. The pancreas could be dissected away from the greater curvature of the stomach and the proximal duodenum with sharp dissection. Hemostasis was maintained using a vessel sealing device (Ligasure, Medtronic, Minneapolis, Minnesota), hemoclips (Hemoclip, Medtronic) and sutures. The pancreatic duct was not identified in any case since none of the resections extended to the level of the duct.

The loop of jejunum used to complete the Roux‐en‐Y procedure was between 15 and 20 cm in length (Figure 5C). The loop of jejunum was harvested from the proximal jejunum 5–10 cm from the duodenocolic ligament at the location where the end‐to‐side anastomosis to restore continuity of the small intestine is easy to perform. If a cholecystojejunostomy (Figure 3) or a choledochojejunostomy (Figure 4) was performed, one jejunal artery was ligated to improve the mobility of the proximal loop of jejunum(Figure 5D). All the end‐to‐side anastomoses during jejunojejunostomy were performed with 4–0 monofilament absorbable sutures (Biosyn, Medtronic) with two simple continuous suture patterns (Figure 5D,E).

The choledochojejunostomies were performed with 6–0 monofilament absorbable suture (Biosyn, Medtronic) with two simple continuous suture patterns. The choledochojejunostomy was performed as an end‐to‐side anastomosis after closing the proximal end of the loop of jejunum with a simple continuous suture pattern with 4–0 monofilament absorbable suture (Biosyn, Medtronic). The end‐to‐side anastomosis was performed 1 cm distal to the blind end of the loop of the jejunum. A 60 mm thoracoabdominal stapler with 4.8 mm staples (TA Stapler, Medtronic) was used to close the pyloric antrum during gastroduodenal resection. For the Billroth I, the duodenum was then anastomosed to the dorsal side of the stomach wall with two simple continuous suture patterns with 4–0 monofilament absorbable suture (Biosyn, Medtronic) in all the cases. In dog 6, the gastrojejunostomy was performed on the ventral side of the stomach since the dorsal side could not be reached because of severe adhesions. The cholecystojejunostomy was completed with two simple continuous patterns with a 4–0 monofilament absorbable suture (Biosyn, Medtronic) as an end‐to‐side anastomosis (Figure 5F–H).

At the time of surgery, gastrostomy tubes were placed in two cats, jejunostomy tubes in one cat and one dog, and jejunostomy through gastrostomy tubes in four dogs. The jejunostomy tubes were advanced in the jejunum aboral to the last anastomosis. No intraoperative complications were recorded in association with the feeding tubes placement. Feeding through the jejunostomy tubes was started immediately after surgery with 25% of the daily caloric requirement and it was increased by 25% every day. Jejunostomy tubes were maintained until the dogs or cats were discharged from the hospital. Gastrostomy tubes were maintained for 9 days (range: 3–78 days). Gastrostomy tubes were aspirated after surgery every 6 h in nine cases. In one case aspiration was continuous. In two dogs, the amount of fluid aspirated from the stomach was 1020 mL (range: 841–1200 mL) in the first 24 h, 532 mL (range: 127–938 mL) in the next 24 h, and 985 mL (range: 87–1884 mL) on day 3 after surgery. In two cats, the amount of fluid aspirated from the stomach was 46.5 mL (range: 45–48 mL) in the first 24 h, 14 mL (range: 7–21 mL) in the next 24 h, and 7 mL (range: 2–12 mL) on day 3 after surgery. Within the population treated for biliary obstruction (6 cases), total bilirubin decreased from 4 mg/dL (0.6–10.6 mg/dL) before surgery to 0.8 mg/dL (0.2–6.1 mg/dL) after surgery. In that same population, ALP decreased from 514.5 IU/L (37–3349 IU/L) before surgery to 255.5 IU/L (33–2418 IU/L) after surgery.

### 3.4 Outcome

One cat and one dog died in the postoperative period of cardiac arrest 5 days and 1 day after surgery, respectively (Table 2). Cat #3 developed septic peritonitis with multiple organ failures 6 days after surgery. An abdominal exploration was performed, and since no dehiscence was observed, the owner elected euthanasia. One dog (dog #4 with a large hepatic mass and severe thrombus in the portal vein) developed septic peritonitis 11 days after surgery. At a second surgery, a dehiscence of the jejunojejunostomy was diagnosed. This dog died of cardiac arrest after the second surgery. Dog #6 developed septic peritonitis 5 days after surgery due to dehiscence of the end‐to‐side jejunojejunostomy. The dog was taken back to surgery, and the Roux‐en‐Y was revised with a new loop of jejunum since after resection of the dehiscence section the first loop of jejunum would have been shorter than 10 cm. A gastrointestinal 60 mm stapler (GIA, Medtronic) and thoracoabdominal 60 mm stapler (TA, Medtronic) with 4.8 mm staples were used for the gastrojejunostomy and the distal jejunojejunostomy. Dog #6 survived the second surgery. A total of 23 anastomoses were performed, resulting in a dehiscence rate of 8.6% (2/23).

Other complications included G‐tube stoma surgical site infections (2/6 cases with gastrostomy tube), hypotension (2/9), and vomiting (2/9). Three cats and one dog required a blood transfusion following surgery. The surgical site infection around the G‐tube did not interfere with the feeding of the dogs and cats. Two dogs and one cat also received plasma in the postoperative period. Postoperatively, dogs were treated with pantoprazole (3), omeprazole (2), ondansetron (2), metoclopramide (4), cisapride (3), maropitant (4), ursodiol (2), and antibiotics (6). Intravenous metoclopramide and cisapride in the gastrostomy tube were used to reduce emesis related to ileus. Postoperatively, cats were treated with pantoprazole (2), omeprazole (5), ondansetron (3), metoclopramide (2), maropitant (1), ursodiol (1), antibiotics (3), and prednisone (1). None of the dogs and cats discharged from the hospital required pancreatic supplementation. In four cases the pyloric antrum or the duodenum were resected, and metoclopramide was required. Metoclopramide was not used in the five cases where the duodenum and the pyloric antrum were preserved.

Four dogs (4/6) and three cats (3/5) were discharged from the hospital (Table 2). The median survival time for the dogs discharged from the hospital was 82 days (range: 60–196 days). The median survival time for the cats discharged from the hospital was 365 days (range: 84–410 days). Of the seven animals discharged, three were euthanized for progressive hepatobiliary disease at 82 days (hepatitis), 196 days (neuroendocrine neoplasia), and 410 days (septic bile peritonitis) after surgery. Two months after surgery dog #6 developed a perforating ulcer in the loop of the jejunum close to the gastrojejunostomy and died of septic peritonitis. At necropsy, a perforated ulcer was diagnosed in the proximal jejunum used for the Roux‐en‐Y. One dog was lost to follow‐up after discharge from the hospital. Two cats were still alive at the time of this study, and according to the owners those cats were not experiencing clinical signs of vomiting or nausea that could have been related to cholangiohepatitis, with one cat being over 1‐year postoperatively.

## 4 DISCUSSION

In this retrospective case series, the Roux‐en‐Y principle was applied in dogs and cats to palliate upper gastrointestinal obstruction or to reconstruct the biliary system. The underlying disease greatly influenced the outcome and owner's decision to pursue any treatment. One dog experienced cholangiohepatitis in the long term due to progression of the neuroendocrine carcinoma that obstructed the bile duct. One dog with chronic hepatitis did not improve after surgery. For the other cases discharged from the hospital, owners did not report any signs of cholangiohepatitis, nausea, or vomiting that are regularly reported with Billroth II or cholecystoduodenostomy in human patients.^4^, ^8^

The principle of the Roux‐en‐Y surgery is the interposition of a loop of jejunum between two other gastrointestinal organs.^4^ This interposed loop can be placed between the gallbladder or the common bile duct and the proximal small intestine (duodenum or jejunum) to prevent reflux of the intestinal contents into the biliary system.^4^ It can also be interposed between the stomach and the rest of the proximal jejunum to prevent biliary reflux into the stomach, ulceration of the gastric mucosa, afferent loop syndrome, and dumping syndrome.^4^ None of those complications were observed in the dogs and cats discharged from the hospital. One dog experienced a perforating ulcer of the jejunal loop between the anastomosis sites; however, this dog had an intrahepatic shunt treated 2 years prior, which has been associated with the development of gastrointestinal ulceration.^12^, ^13^ Ulceration of the Roux‐en‐Y loop in this case was suspected to be secondary to the intrahepatic shunt rather than the Roux‐en‐Y procedure.

The length of the loop of jejunum for the Roux‐en‐Y in this study was empirically chosen between 15 and 20 cm for all cases. In human patients, using a segment 40–70 cm long has been recommended to reduce the Roux stasis.^4^ Such length could not be used in dogs or cats because it would have represented more than half of the jejunum in most cases. The ideal length of the jejunum loop for Roux‐en‐Y is unknown. The Roux stasis results from disruption of peristaltic wave in the harvested segment of jejunum and it results in accumulation of gastrointestinal content that can result in nausea and vomiting.^4^ The loops of jejunum used in the Roux‐en‐Y are harvested from the proximal jejunum. In human patients, the ligament of Treizt is the landmark to harvest the proximal loop of jejunum.^4^ In this study on dogs and cats, the duodenocolic ligament was used as a landmark, and the loop of jejunum was isolated at a location aborad to the duodenocolic ligament which was easy to reach without transecting the ligament. When the jejunum was mobilized to reach the gallbladder, most cases required ligation of one branch of the jejunal artery to improve mobilization of the proximal end of the loop of jejunum. It has been shown that three mesenteric arteries can be ligated from a pedicle jejunal autograft without affecting the blood supply to the harvested loop of the jejunum without additional surgical intervention.^14^

The decision to use any of the four configurations of the Roux‐en‐Y was made during surgery. Figures 1, 2, 3, 4 represent the variations possible with the Roux‐en‐Y principle. The techniques described in Figures 1, 2, 4 were used in combination with a Billroth I when the biliary system was affected by the resection of the duodenum or when the distal common bile duct had to be resected to remove a tumor. The technique described in Figure 1 was used only once. The technique described in Figure 2 was used to eliminate the need for end‐to‐end anastomosis in the jejunum in Figure 1. The proximal end of the loop of the jejunum was anastomosed to the gallbladder, and the proximal jejunum was anastomosed with an end‐to‐side anastomosis 15–20 cm distal to the cholecystojejunostomy. The technique in Figure 2 eliminates an extra end‐to‐end anastomosis in the jejunum, more likely reducing the risk of leakage and dehiscence. A choledochojejunostomy was performed if the common bile duct was dilated and sufficient length was present (Figure 4). If the common bile duct was either too short or not dilated, then a cholecystojejunostomy (Figure 2) was used. A choledochojejunostomy (side to side or end to side) is the technique of choice in human patients for better long‐term outcomes because it provides another barrier to reduce the risk of ascending infection into the biliary tract compared to a cholecystojejunostomy.^15^, ^16^ Also, the choice between a cholecystojejunostomy and a choledochojejunostomy depends on the ability of the surgeon to perform any of those procedures. The technique described in Figure 3 was used when a Billroth II would have been needed because the proximal duodenum could not reach the pyloric antrum or the body of the stomach.

The Roux‐en‐Y procedure was associated with high morbidity in this study. Septic peritonitis developed in two dogs and one cat after surgery. All three cases went to surgery for a second abdominal exploration. A dehiscence rate of 8.6% was present in our study, which seems lower than currently reported for gastrointestinal anastomosis when compared to a cholecystojejunostomy.^17^ In the future, the utilization of stapling equipment might reduce the risk of dehiscence since cases requiring a upper gastrointestinal resection may have several risk factors for dehiscence.^17^, ^18^, ^19^ Postoperatively, nausea and vomiting were the most common complications but were self‐limiting and resolved by the time the animals were discharged. Nausea and vomiting were also suspected to be related to severe ileus present after upper gastrointestinal reconstruction, presence of septic peritonitis, and the administration of opioids for pain control. The Roux‐en‐Y procedure has been shown to delay gastric emptying by interfering with the migration of contraction across the stomach and the loop of jejunum and because of the removal of the pyloric antrum and or the duodenum.^20^, ^21^ Also, the length of the segment of the jejunum interposed affects the propagation of peristaltic waves and gastric emptying and may worsen the Roux stasis syndrome.^4^, ^22^ In this study, intravenous metoclopramide was used in cases requiring resection of the pyloric antrum and/or the duodenum. Pantoprazole and omeprazole were used to help control nausea, vomiting, and gastric ulceration in most of the cases in this study.

Gastrostomy tubes were used to decompress the stomach the first 3 days after surgery and reduce the risk of regurgitation, vomiting, and nausea. The amount of fluid withdrawn from the stomach decreased over the first 3 days after surgery, likely because the postoperative ileus was resolving. Gastrostomy tubes were chosen over esophagostomy tubes and nasogastric tubes because gastric decompression was necessary while ileus was present and because of the need for long‐term nutrition. The gastrostomy tubes were maintained for a minimum of 2 weeks after discharge from the hospital. The jejunostomy feeding tubes were used immediately after surgery to help provide caloric support and protein to the dogs and cats and to bypass the surgical site. Since the Roux stasis syndrome can affect the motility of the interposed loop jejunum, the authors have the impression it is important to feed into the jejunum distal to the last anastomosis, especially for the construct in Figure 1. Mild to moderate hypoalbuminemia was present in all the dogs and cats after surgery in this study. Plasma transfusions were administered in two cases where post‐operative albumin decreased below 2 mg/dL. Cisapride and omeprazole were required in the long term for all the dogs and cats discharged from the hospital to stimulate peristalsis and treat the presumed gastritis that was present prior to upper gastrointestinal reconstruction. None of the dogs and cats discharged from the hospital required pancreatic supplementation even after biliary reconstruction. For all cases discharged from the hospital, the clinical signs observed before surgery were absent at discharge.

None of the cases with cholecystojejunostomy or choledochojejunostomy developed bile leakage after surgery. Choledochojejunostomy is a problematic part of the procedure, with a high rate of bile leakage and stricture formation in human patients.^10^, ^23^ In this study, choledochojejunostomies were performed with common bile ducts that were very dilated. The similarity in luminal size between the dilated common bile ducts and intestinal lumen of cats and dogs may have contributed to the improved outcomes of the choledochojejunostomy compared to humans.^10^, ^23^

The mortality rate associated with this population of dogs and cats undergoing a Roux‐en‐Y procedure was high at 36%. Septic peritonitis and cardiac or respiratory arrest in the postoperative time were the most common causes of death in this study because they were very hemodynamically unstable or were advanced in their underlying conditions with a guarded prognosis. Most of the surgeries were performed in dogs and cats with severe gastrointestinal or biliary disease, which likely affected the outcome of the procedure. The Roux‐en‐Y procedure requires several gastrointestinal anastomoses, which increases the risk of leakage or dehiscence regardless of the animals' health status.

Limitations of this study include the small population with variable presentations, and the retrospective nature of this report. Also, there is a large spectrum of underlying diseases present in this study, making it difficult to make specific recommendations regarding indications and best practices. Regardless, the Roux‐en‐Y procedure has been shown to palliate many of the complications related to Billroth II and cholecystoduodenostomy in human patients.^4^ Due to the retrospective nature of the study long term complications are likely under reported.

Roux‐en‐Y procedure could be considered when an upper gastrointestinal reconstruction is needed in dogs and cats. Motility disorders affecting gastric emptying should be anticipated and addressed in the postoperative period.

## AUTHOR CONTRIBUTIONS

Fink B, DVM, MS: Reviewed the medical records, analyzed the data, and wrote the manuscript. Marvel S, DVM, MS, DACVS (Small Animal): Helped analyze the data and edited the manuscript. Monnet E, DVM, PhD, DACVS, DECVS: Reviewed the data, helped analyze the data, and edited the manuscript. All authors provided a critical review of the manuscript and endorsed the final version. All authors are aware of their respective contributions and have confidence in the integrity of all contributions.

## CONFLICT OF INTEREST STATEMENT

The authors declare no conflicts of interest related to this report. This data has not been presented in any meeting.
